# Formal Thought Disorder and Executive Functioning in Children and Adolescents with Autism Spectrum Disorder: Old Leads and New Avenues

**DOI:** 10.1007/s10803-017-3104-6

**Published:** 2017-03-24

**Authors:** Tim Ziermans, Hanna Swaab, Alexander Stockmann, Esther de Bruin, Sophie van Rijn

**Affiliations:** 1 Department of Clinical Child and Adolescent Studies, Leiden University, Wassenaarseweg 52, Leiden, 2333 AK The Netherlands; 2 Leiden Institute for Brain and Cognition, Leiden University, Leiden, The Netherlands; 3 Autism Center Rivierduinen, Leiden, The Netherlands; 4 Research Institute Child Development and Education, University of Amsterdam, Amsterdam, The Netherlands; 5 Research Priority Area Yield, University of Amsterdam, Amsterdam, The Netherlands; 6 UvA minds, Academic Outpatient Child and Adolescent Treatment Center, Amsterdam, The Netherlands

**Keywords:** ASD, High-functioning, Thought disorder, Executive functioning, Working memory, Psychosis

## Abstract

Formal thought disorder (FTD) is a disruption in the flow of thought and a common feature in psychotic disorders and autism spectrum disorder (ASD). Executive dysfunction has often been associated with FTD, yet for ASD convincing evidence is lacking. This study investigated FTD and three core executive functions in 50 young children and adolescents with high-functioning ASD and 56 matched controls. Higher overall levels of FTD marked ASD compared to controls. Furthermore, in ASD decreased performance on verbal working memory was correlated with increased FTD ratings and explained a significant amount of variance of objective and subjective FTD. Verbal working memory is currently the most promising target executive function for understanding the development of idiosyncratic thought disorders in ASD.

**Electronic supplementary material:**

The online version of this article (doi:10.1007/s10803-017-3104-6) contains supplementary material, which is available to authorized users.

## Introduction

Autism spectrum disorders (ASD) and psychotic disorders, such as schizophrenia, both represent severely disabling neurodevelopmental disorders (Goldstein et al. 2002), with marked impairments in cognition and social functioning (Couture et al. 2010; Sasson et al. 2011). For ASD, the first behavioral problems typically occur during early childhood, while psychotic disorders are primarily diagnosed in late adolescence and young adulthood. Despite some symptomatic overlap, both psychiatric classifications are largely characterized by differential behavioral phenotypes and appear mutually exclusive in diagnostic manuals such as the *Diagnostic Statistical Manual of Mental Disorders* (5th edition; DSM5; American Psychiatric Association 2013) and the *International Classification of Diseases* (10th edition; ICD-10; World Health Organization 1993). Interestingly though, there is substantial evidence indicating that children diagnosed with ASD have an increased prevalence of psychotic disorders later in life and that psychotic disorders are associated with increased rates of ASD (Chisholm et al. 2015; Selten et al. 2015).

In recent years there has been a renewed interest in specific overlapping autistic and psychotic symptoms, in clinical and non-clinical samples, partially guiding the ongoing quest for differential markers and potential risk factors for psychosis in patients with ASD (Abu-Akel et al. 2016, 2015; Barneveld et al. 2011; Brosnan et al. 2014; Chung et al. 2014; Crespi et al. 2016; Eack et al. 2013; Sasson et al. 2016). One overlapping symptom that has received relatively little attention is formal thought disorder (FTD). FTD refers to a disruption in the flow of thought, as observed by disorganized speech. Although speech is an indirect measure of thought, “*language serves essentially for the expression of thought*”, as phrased by the famous linguist Noam Chomsky (Chomsky et al. 1979), whose extensive work in this field implies that an understanding of the rules of a language throws light on the principles that regulate human thought.

FTD represents a hallmark feature of schizophrenia and is also characteristic of childhood onset schizophrenia, schizotypal personality disorder (Caplan 1994a), and predictive of psychosis in adolescents at clinical high-risk (Bearden et al. 2011). Likewise, speech disturbances, such as pragmatic language impairments, are a common feature in ASD. Obviously, it requires a minimal amount of speech in order to tap into the organization of thought in terms of logic and coherence. Indeed, when assessing FTD in verbal, high-functioning individuals with ASD there is some evidence of increased levels of FTD. Earlier studies in small adult samples of high-functioning individuals reported that, compared to adults with schizophrenia, those with ASD demonstrated more ‘negative’ thought disorder, i.e. poverty of (content of) speech, but not so much ‘positive’ features, i.e. illogicality and derailment (Dykens et al. 1991; Rumsey et al. 1986). More recently, elevated rates of FTD have also been reported in children with high-functioning ASD (Solomon et al. 2008; van der Gaag et al. 2005). These studies did report elevated rates of ‘positive’ FTDs (i.e. ‘illogical thinking’ and ‘loose associations’) in ASD compared to typically developing children. This is relevant, because this may point to disorganization of thought in a way that may predispose to symptoms that are typically observed in individuals with psychotic disorders.

There currently is no evidence suggesting that FTD is indicative or predictive of a subsequent psychotic episode in children with high-functioning ASD (Eussen et al. 2015; van der Gaag et al. 2005), although this has never been investigated in a direct manner. However, one long-term clinical follow-up study of a group of 55 ASD children meeting criteria for multiple complex developmental disorder (MCDD), a descriptive ASD subtype marked specifically by deregulation of thought and emotions (Buitelaar and van der Gaag 1998; Sprong et al. 2008; van der Gaag et al. 1995), reported that approximately 70% of participants met criteria for schizophrenia spectrum disorder in adolescence or adulthood (van Engeland and van der Gaag 1994). As such, it remains uncertain to what extent early observation of FTD may contribute to the development of psychotic disorders in later life.

In addition to a clear link with impaired semantic processing skills, there is a seemingly strong association between impaired executive functioning and FTD in patients with schizophrenia (Docherty 2012; Kerns and Berenbaum 2002). The term ‘executive functions’ refers to a set of cognitive processes associated with the control of thoughts and actions (Bunge and Souza 2009). Executive functions include, but are not limited to, cognitive abilities such as response inhibition, working memory/updating, and set shifting (Friedman and Miyake 2016; Miyake et al. 2000). It is well established that executive functions are impaired along the full width of the psychosis spectrum (e.g. Bora and Murray 2014; Giakoumaki 2012; Ziermans 2013), although results have been mixed concerning their added use for predicting psychotic onset on an individual level (Fusar-Poli et al. 2012; Lin et al. 2013; Metzler et al. 2016; Ziermans et al. 2014). Executive dysfunction has also been historically linked to ASD (Pennington and Ozonoff 1996) and impairments have been widely reported (for a review see Russo et al. 2007), albeit within the context of large individual and age-dependent differences (Pellicano 2010; van den Bergh et al. 2014). Such individual differences in ASD may also partially account for the observed symptomatic overlap with psychotic disorders, but only a limited amount of studies have addressed this issue.

Only one pilot-study has previously investigated FTD in relation to executive functioning in ASD (Solomon et al. 2008). Solomon and colleagues compared 17 adolescents with high-functioning ASD to 21 matched controls on objective rating scales for FTD and examined correlations with one executive control task (Preparing to Overcome Prepotency; POP-task; Barber and Carter 2005), which measures inhibition of a prepotent response. They found that only one type of FTD (illogical thinking) was specifically correlated to response inhibition with borderline significance. However, sample sizes were small and this study focused specifically on inhibition, which limits the ability to firmly establish which aspect of EF is most relevant for understanding FTD.

The first goal of the current study was to compare the prevalence of FTD, as measured by both objective ratings and subjective self-reports, between a substantial group of high-functioning children and adolescents with ASD and their typically developing controls (TDC), matched for age, gender and (verbal) IQ. Based on two previous reports in smaller samples, we expected that children and adolescents with ASD would show higher levels of FTD, in particular for ratings of illogical thinking and loose associations (Solomon et al. 2008; van der Gaag et al. 2005). Second, we aimed to determine whether level of FTD was impacted by cognitive performance on three core executive functions (response inhibition, working memory and cognitive flexibility) in high-functioning ASD. Establishing the relative contribution of executive functions to FTD, in the context of relatively preserved verbal skills, can potentially lead to improved early identification of cognitive risk factors for the development of psychotic symptoms in ASD, as well as provide new incentive for fine-tuning research into the underlying neurodevelopment of both types of disorders. We expected to find multiple associations between executive functions and FTD parameters and, more specifically, that response inhibition measures would explain a significant amount of variance in FTD (Barneveld et al. 2013; Solomon et al. 2008).

## Methods

### Participants

The ASD group was recruited from a child psychiatric outpatient department with specialized services for children with autism (Autism Center Rivierduinen), serving a large region in the Netherlands. All children with ASD were classified according to the Diagnostic and Statistical Manual of Mental Disorders, Fourth Edition (DSM-IV; American Psychiatric Association, 1994) criteria and were considered to be high-functioning (IQ ≥ 70). The clinical procedures for diagnosis of ASD included questionnaires for parents, an interview with parents (Autism Diagnostic Interview-Revised; Le Couteur et al. 2003; ADI-R), developmental history and family history, information from treating physicians and extensive expert clinical observations. Consensus regarding the diagnostic classification of ASD had to be reached by board-certified child psychiatrists (with experience in the field of autism) and by a consensus meeting with a multidisciplinary team.

Typically developing controls (TDC) were recruited from schools distributed across the western part of the Netherlands and screened for psychopathology: none scored in the clinical range (T ≥ 70) on the diagnostic subscales of the Childhood Behavior Checklist (CBCL; Achenbach 1991). Inclusion criteria for all participants were: Dutch as the primary language and aged between 9 and 19 years. Exclusion criteria were a recent history of substance abuse, intellectual disability (diagnosed or IQ < 70) and neurological conditions. After providing all necessary information about the study to the subjects and their parents, written informed consent was obtained, in accordance with the Declaration of Helsinki. The Ethical Committee of Leiden University Medical Center, the Netherlands, approved this study.

### Descriptive Measures

#### Intellectual Functioning

The subtests Block Design and Vocabulary of the Dutch adaptations of the third edition of the Wechsler Intelligence Scales for Children (WISC-III-NL; Kort et al. 2002) were used to assess IQ, i.e. the Vocabulary-Block Design (V-BD) short form. This form is frequently used to estimate full-scale IQ (FSIQ) according to the algorithm (2.9 × [sum of normed scores] + 42) (Campbell 1998). The V-BD short form correlates highly with FSIQ (*r* = .88) (Herrera-Graf et al. 1996), and has been found valid for the estimation of intelligence, with a good reliability (*r* = .91) and validity (0.82) (Campbell 1998).

#### Autism Traits

The Autism spectrum Quotient - Children’s version (AQ-Child; Auyeung et al. 2008) is a parent questionnaire of 50-items that can reliably assess the degree to which an individual might have features of the core autistic phenotype. The AQ-Child is an adaptation of the self-report version (Baron-Cohen et al. 2001). For the Dutch versions only the self-report version has been validated (Hoekstra et al. 2008), and the parent report is a similar adaptation for children as for the English versions. Five subscales cover personality traits associated with the autism spectrum; social skills, communication, imagination, attention to detail, and attention switching. Binary scoring was used for all items with a maximum score range of 0–50. Higher scores on the AQ indicate higher levels of autism traits.

### Thought Disorder Measures

#### The Kiddie-Formal Thought Disorder Rating Scale

The golden standard assessment for FTD in children is the Kiddie-Formal Thought Disorder Story Game procedure with its accompanying rating Scale (K-FTDS), a frequently used and reliable, objective measure of FTD in children ≥7 years (Caplan 1994b; Caplan et al. 2000). In the first and third part, the child listened to an audiotaped story: (1) the first story is about a boy dreaming about a friendly ghost and the third story is about a boy who is excluded from his group of friends and badly teased. Children were asked standard questions, for instance: “What did you like about this story?” In the second part of the story game, the child was asked to make up his or her own story chosen from four topics: (a) the horrible hulk (b) a witch, (c) a disobedient child or (d) an unhappy child. Assessments lasted for about 20–30 min and speech samples were recorded using a digital audio recorder.

Four different subtypes of FTD were coded according to the guidelines by Caplan (1994b): Illogical Thinking (ILL); Loose Associations (LA); Poverty of Content (POC) and Incoherence (INC). Utterance counts for the total speech sample were also calculated. There were two independent raters who were never the same person as the interviewer. Both received training from author EdB, who was formally trained in K-FTDS assessment and rating procedures by Caplan. The raters were blind to group membership and diagnosis. Inter-rater reliability was maintained through regular consensus meetings in which the independent coding of all participants by both raters was discussed. The raw error scores were divided by the number of utterances to correct for variance in total amount of speech produced and to calculate the number of errors per minute. In addition, a total sum score was also calculated (Total FTD), and available cut-off scores (Caplan et al. 1989) for optimal sensitivity and specificity were used to create dichotomized scores for ILL, LA and Total FTD. Scores above the cut-off point reflect a higher likelihood of pathology. Caplan and colleagues were unable to calculate reliable cut-off scores for POC and INC due to infrequent ratings for these subscales.

#### Schizotypal Personality Questionnaire—Odd Speech

Although K-FTDS outcomes are considered an objective measure of FTD, its training and rating procedures are also very time consuming and difficult to obtain during clinical assessment (de Bruin et al. 2007). Therefore it is worth investigating alternative, more subjective measures of FTD, which can be more readily assessed. The Schizotypal Personality Questionnaire (SPQ; Raine 1991) is a self-report questionnaire assessing schizotypal traits. For this study we used the Odd Speech subscale of a previously validated Dutch translation, adjusted for children (SPQ-C-D; van Rijn et al. 2015). Odd Speech is thought to reflect disorganized thought of the individual and together with the Eccentric/Odd Behavior subscale constitutes the Disorganization dimension of the SPQ. The subscale consists of nine items, which ask about subjective and external qualifications about the individual’s speech. For example, ‘People sometimes find it hard to understand what I am saying’, or ‘I sometimes use words in unusual ways’. All items are answered with ‘correct’ (i.e. applies to me) or ‘incorrect’ (i.e. does not apply to me) and receive a binary score, based on which a sum score is calculated (range 0–9).

No formal clinical cut-off score is available for the Odd Speech subscale; therefore we used data from our validation sample of 219 typically developing children and adolescents (van Rijn et al. 2015) to establish a proxy for a clinical cut-off score. A T-score of 67 is a commonly used borderline score on clinical questionnaires, which is equivalent to a score within the 95th percentile. The 95th percentile for the Odd Speech subscale referred to a score of 8, which was used as a clinical cut-off to create a dichotomous variable for the purpose of this study.

### Executive Functioning Measures

Three computerized tasks from the Amsterdam Neuropsychological Tasks (ANT, version 2.0; de Sonneville 2005) and one subtest (number repetition) of the Dutch version of the Clinical Evaluation of Language Fundamentals (CELF-IV; Kort et al. 2008) were included in this study. The ANT has been used extensively to examine executive functions and related cognitive processes in various clinical and non-clinical populations and has high sensitivity for neuropsychological dysfunction as well as good reliability and validity (de Sonneville 2014). All computer tasks were preceded by instructions of the test leader and practice trials. Number repetition was administered following CELF manual guidelines.

#### Working Memory

Visuospatial working memory was measured with the ANT Spatial Temporal Span (STS). In this task a gray square containing nine smaller squares positioned on a 3 × 3 matrix is visible on the screen. After presentation of an auditory cue a hand animation is run and successively points at a number of these nine squares (1000 ms stimulus presentation, 750 ms to move hand to the next stimulus). Children are instructed to remember the locations (ranging from 2 to a maximum of 9) and after the stimulus is presented indicate the locations by clicking on them in the order they appeared on the screen (i.e., the correct temporal order). The task consists of two parts of a maximum of 24 trials: forward span and backward span. In both parts the task automatically ends after two consecutive errors of the same type. The number of correctly completed trials in the correct order for the backwards condition was used as the variable of interest in this study.

Verbal working memory was assessed with the digit span subtest ‘number repetition’ of the CELF (CELF-NR), which also contains a forward and backward condition. In this subtest children are asked to repeat orally presented strings of numbers (which increase in size) in either the correct (forward) or reverse order (backward). Total number of correct items backwards was used for the analyses.

#### Response Inhibition

The ANT Go-NoGo (GNG) task was used to measure the capacity to inhibit a prepotent response. Stimuli consisted of ‘Go’-stimuli (gray square with yellow frame) and ‘NoGo’-Stimuli (same as Go, with a small spatial gap at the bottom of the frame). Children were instructed to click a mouse button as quickly and accurately as possible when a Go-stimulus was presented. If the NoGo-stimulus was presented, the subjects were instructed to withhold clicking the button. The stimulus was presented for 300 ms. The valid response window was 200–1500 ms post onset of the stimulus. Stimuli were pseudo-randomly presented (biased condition: 56 Go-stimuli and 18 NoGo-stimuli) to measure inhibition of an ongoing response. Variables of interest were speed (reaction time Go-signals) and accuracy (d’: *Z*(hit rate) − *Z*(false alarm rate)).

#### Cognitive Flexibility

The ANT Shifting Set Visual (SSV) task was used to measure cognitive flexibility of participants. During this task a green or red colored square jumps randomly to the right or left on a horizontal bar of ten gray squares. Depending on the color of the square, the participant has to execute a compatible (pressing the key in the same direction) or an incompatible response (opposite direction). The task is self-paced with a response window of 150–5000 ms. The test consists of three parts: the first part requires compatible responses to 40 left/right jumps of the green square, the second part requires 40 incompatible responses to a jumping red square and for the third part the color of the square varies between red and green (random condition). The third part consists of 80 trials (40 compatible, 40 incompatible) and flexibility can be measured by contrasting part one and the compatible condition of part three on speed and accuracy.

### Data Analysis

Data were analyzed with IBM SPSS version 22. Baseline characteristics of the control and training group were compared with Chi square and independent t tests or non-parametric Mann–Whitney *U* tests for test variables with non-normal distributions. Cohen’s *d* was calculated based on pooled group variances (*M*_1_ − *M*_2_/*s*_pooled,_ where *s*_pooled_ = √[(*s*_1_^2^ + *s*_2_^2^)/2]) to determine the effect size of group mean differences. Two-tailed Spearman’s rank correlations were performed to check for any significant associations between dependent and independent variables in the ASD group. Next, regression analyses were used to investigate whether performance on executive tasks could predict the level of thought disorder in ASD. First by using linear regression with continuous dependent variables and second by means of logistic regression and binary dependent variables (based on cut-off scores). To check for the potential influence of age, sex and medication use (binary), all regression analyses were repeated with these background variables entered as covariates. Finally, to explore whether autistic traits were moderating any relations between EF and FTD, regression analyses were repeated with mean-centered total AQ scores and its interaction-term with relevant EF-variables entered into the model. Alpha for significant effects was set at *p* < .05.

## Results

### Participants

The total participant group consisted of 50 children and adolescents with ASD (41 boys, 9 girls) and 56 TDC (47 boys, 9 girls). To prevent unequal gender distributions, female control participants were selected from a large pool of control girls and individually matched to ASD girls based on age and FSIQ scores. For five ASD individuals we were unable to conduct the ADI-R parent-interview. Of the remaining 45 individuals, 34 (75.6%) scored above cut-off on all three ADI-R domains and an additional 9 (20%) scored above cut-off on two domains. ADI data was used in the diagnostic evaluation; all ASD individuals received a formal diagnosis of ASD after a thorough clinical evaluation procedure, described in the “Methods” section above. Further group characteristics are displayed in Table 1. Groups did not differ on age and IQ scores, and TDC showed significantly fewer ASD symptoms on all AQ scales. Finally, five ASD individuals (3 boys, 2 girls) received psychotropic medication.

**Table 1:** Group characteristics

	ASD (*n* = 50) *M* ± *SD*	Range	Controls (*n* = 56) *M* ± *SD*	Range	Statistic	*p*
Age	12.23 ± 2.21	9.0–18.2	12.40 ± 2.97	9.1–19.0	*t* = 0.35	.730
FSIQ	102.55 ± 14.57	71–129	101.08 ± 12.81	74–135	*t* = 0.56	.580
Verbal (vocabulary)	10.14 ± 2.94	4–16	10.51 ± 2.72	5–18	*t* = 0.56	.506
Performal (block design)	10.73 ± 3.17	2–16	9.86 ± 2.98	3–18	*t* = 0.56	.107
AQ (total)^a^	29.98 ± 9.36	11–44	13.21 ± 6.51	3–27	*U* = 2170.5	<.001
Social skill	6.26 ± 2.86	1–10	1.78 ± 1.57	0–5	*U* = 2126.0	<.001
Attention switching	7.23 ± 1.94	3–10	2.96 ± 1.97	0–8	*U* = 2192.5	<.001
Attention to detail	5.02 ± 2.22	1–10	3.96 ± 2.02	0–10	*U* = 1499.5	.022
Communication	6.33 ± 2.36	1–10	2.22 ± 2.11	0–8	*U* = 2105.5	<.001
Imagination	5.14 ± 2.56	1–10	2.49 ± 1.67	0–8	*U* = 1892.0	<.001

^a^AQ data was missing for 1 control and 7 ASD individuals

### Formal Thought Disorder

K-FTDS data was missing for three individuals with ASD and SPQ-C-D data for two individuals with ASD. All data distributions were skewed and there were no extreme outliers. Consequently, analyses were performed with non-parametric Mann–Whitney *U* tests and group comparisons for K-FTDS and Odd Speech are listed in Table 2.

**Table 2:** Group comparisons for thought disorder measures

	ASD *M* ± *SD*	Range	Controls *M* ± *SD*	Range	Statistic	*p*	d
K-FTDS							
Utterances per minute	10.66 ± 2.70	5.00–16.40	10.34 ± 2.75	2.00–15.40	*U* = 1357.0	.786	0.12
Ratings for Total FTD	0.13 ± 0.12	0.00–0.48	0.09 ± 0.12	0.00–0.63	*U* = 1609.0	**.044**	0.31
Ratings for illogical thinking	0.10 ± 0.10	0.00–0.43	0.08 ± 0.12	0.00–0.63	*U* = 1372.0	.121	0.15
Ratings for loose associations	0.01 ± 0.04	0.00–0.23	–	–	*U* = 1535.5	.128	0.27
Ratings for poverty of content	–	–	–	–	NA	NA	NA
Ratings for incoherence	0.02 ± 0.06	0.00–0.36	0.00 ± 0.02	0.00–0.14	*U* = 1461.0	**.050**	0.09
SPQ							
Odd speech	4.53 ± 2.72	0–9	2.68 ± 2.46	0–9	*U* = 1902.0	**<.001**	0.71

K-FTDS ratings represent errors divided by utterances per minute

Bold = *p* < .05

*NA* not applicable

#### K-FTDS

Participants with ASD showed significantly higher rates of FTD as measured by Total FTD and INC, but not for any of the other subdomains. Effect sizes were small for all variables (*d*_*range*_ = 0.09–0.31). Percentages of cases scoring above clinical cut-off indicated that in absolute terms more individuals with ASD than TDC scored in the clinical range on K-FTDS total score (25.5 vs. 14.3%), ILL (46.8 vs. 28.6%) and LA (4.3 vs. 0.0%).

#### Odd Speech

Individuals with ASD reported significantly more FTD than TDC and the effect size was medium (*d* = 0.71) according to Cohen’s definition of effect sizes. Percentages of cases scoring above the cut-off indicated that more children and adolescents with ASD than TDC reported elevated levels of subjective FTD (16.3 vs. 7.1%).

### Executive Functioning

Working memory data (STS and CELF-NR) was missing for one individual with ASD. For the response inhibition task (GNG) data was missing for three TDC and one participant with ASD. In addition, two outliers (controls) with a false alarm rate >90% were removed for analysis. Data on cognitive flexibility was missing for one TDC and one individual with ASD. Except for speed parameters (GNG and SSV) data were not normally distributed.

#### Correlations with Age, IQ and Autism Symptoms

Correlations are displayed in Table 3 for the ASD group only. Both working memory measures and both inhibition measures were significantly correlated with each other (CELF-NR–STS: *r* = .36, *p* = .011; GNG speed/accuracy: *r* = −.35, *p* = .015). STS was also significantly correlated to GNG (speed: *r* = −.38, *p* = .008; accuracy: *r* = .43; *p* = .002). Concerning background variables, all EF measures showed increased performance with age, although this did not reach statistical significance for CELF-NR and SSV (accuracy). Furthermore, CELF-NR, STS and SSV (accuracy) were positively correlated with FSIQ (all *p* < .05, STS *p* < .01). These correlations were mainly driven by performance on the performal subtask and not correlated with verbal skills. There were also no significant correlations between executive functioning and AQ. Correlations for the total group including controls are available in the Supplemental Table.

**Table 3:** Pairwise Spearman correlations of executive functions with global group characteristics and FTD in the ASD group

	1	2	3	4	5	6	7	8	8a	8b	9	10	11	12
Executive functioning														
1. Verbal working memory														
2. visuo-spatial working memory	**.360**													
3. Response inhibition (speed)	−.212	−.375*												
4. Response inhibition (accuracy)	.236	.426*	**−.347**											
5. Cognitive flexibility (speed)	.085	−.237	.192	−.179										
6. Cognitive flexibility (accuracy)	−.036	**−.305**	.207	−.152	.228									
Group Characteristics														
7. Age	.118	.430*	−.369*	**.321**	−.250	−.435*								
8. FSIQ	**.305**	.490*	−.208	−.254	−.161	**−.306**	.036							
8a. Verbal	.199	.199	−.088	.056	−.134	−181	−.012	.825*						
8b. Performal	**.332**	.617*	−.268	**.354**	−.157	**−.333**	.097	.829*	.381*					
9. AQ—total	−.247	−.166	−.043	−.020	−.252	−.221	−.001	−.047	.007	−.073				
Formal thought disorder^a^														
10. K-FTDS—total FTD	−.242	−.175	.205	−.140	−.012	−.123	−.181	−.165	−.136	−.155	.103			
11. K-FTDS—illogical thinking	−.164	−.102	.106	−.054	−.047	−.162	−.168	−.157	−.147	−.119	−.032	.904*		
12. K-FTDS—incoherence	−.238	−.070	.265	−.214	.004	−.049	−.009	−.122	−.091	−.162	.257	.448*	.167	
13. SPQ—odd speech	−.400*	−.183	.135	−.080	.134	.031	−.231	−.133	−.089	−.166	.029	.226	.268	.180

Bold = *p* < .05; *p < .01

^a^K-FTDS ratings represent errors divided by utterances per minute; subscales for loose associations and poverty of content were not included due to low frequencies

### Regressing Executive Functions on Formal Thought Disorder in ASD

To minimize the number of predictor variables, only executive functioning measures correlating with FTD measures were entered in linear regression models, and for logistic regression models only the four highest correlating variables were entered as predictors (*n.b*. logistic regression does not assume a linear relationship between dependent and independent), allowing for a ratio of at least ten cases per predictor (See Table 3 for correlations). In addition, a forward stepwise method (Likelihood Ratio) was used for logistic regression. Although all assumptions for both types of regression analyses were met, it is worth noting that residuals indicated limited homoscedasticity in general. No influential cases were detected.

#### Linear Regression Models

There was one significantly correlated variable-pair: CELF-NR and SPQ-Odd Speech; K-FTDS measures did not have linear relationships with executive functions (Table 3). In the regression analysis CELF-NR significantly predicted Odd Speech (*R* ^*2*^ = 0.11; β = −0.37, *t* = −2.71, *p* = .009) and remained significant after adjusting for age, sex and medication (*R* ^*2*^ = 0.11; β = − 0.33, *t* = −2.29, *p* = .027) and there was no significant moderating effect of autism traits (CELF-NR × Total AQ: *p* = .30). The negative beta indicated that a worse performance on verbal working memory was associated with increased levels of (subjective) FTD in children and adolescents with ASD.

#### Logistic Regression Models

Models were run for two dependent variables; binary variables for Total FTD and Odd Speech. Both variables correlated highest with the four variables for working memory and response inhibition. Regression results showed that for both the subjective and objective FTD measures CELF-NR was the only significant predictor in the final model, with odds ratios indicating that better WM performance decreases the odds of scoring above clinical threshold. When age, sex and medication were forced into the model with significant predictors, both the model and CELF-NR backwards remained unchanged in terms of significance. However, none of these background variables contributed significantly to the model, which rendered their inclusion unnecessary. Furthermore, no significant interactions of CELF-NR with total AQ were detected (*p* = .20 and *p* = .24, respectively). Parameters for both univariate models are displayed in Table 4.

**Table 4:** Univariate models of executive functions predicting clinical level of formal thought disorder

Model^a^	B	SE	Wald	*p*	Odds ratio	95%CI
K-FTDS-total FTD						
Constant	−1.44	0.46	10.00	.002	0.24	
Verbal working memory (CELF – NR)	−1.18	0.57	4.30	.038	0.31	0.10–0.94
SPQ-odd speech						
Constant	−2.72	0.81	11.19	.019	0.06	
Verbal working memory (CELF – NR)	−2.25	0.94	5.70	.001	0.11	0.02–0.67

^a^Logistic regression with forward stepwise elimination—final models (p < .05)

## Discussion

The findings in the current study suggest that children and adolescents with high-functioning ASD experience elevated levels of FTD, both objectively and subjectively, even in the context of intact (verbal) intellectual functioning. This corroborates previous accounts of increased rates of FTD in children and adolescents with high-functioning ASD (Solomon et al. 2008; van der Gaag et al. 2005). Few studies have investigated putative cognitive mechanisms driving these observed difficulties in the organization of thought and speech in ASD. Executive functions represent a set of interrelated cognitive skills that allow people to exercise a certain amount of control over their thoughts. These cognitive functions are often impaired in ASD and sometimes linked to FTD in schizophrenia spectrum disorders (Barrera et al. 2005; Docherty 2012). As such, we hypothesized that impaired executive functioning, in particular inhibitory control, would predict levels of FTD in ASD as well. Our study showed that, when evaluating multiple executive functions, verbal working memory was the single one associated with FTD.

Increased presence of FTD may or may not predispose adolescents for psychotic disorders. Evidence from a clinical high-risk population (CHR), presumably without ASD diagnosis, suggests that higher ratings for illogical thinking and poverty of content can help predict the onset of psychosis and level of social functioning approximately one year after intake (Bearden et al. 2011). The only known follow-up study that investigated predictive validity of FTD in high-functioning ASD reported that illogical thinking in young children did not predict prodromal symptoms in adolescence 7 years later, and instead was more indicative of ASD symptom severity (Eussen et al. 2015). However, the outcome measure consisted of a screening questionnaire for prodromal symptoms, assessed in children aged 12–20 years, so before the peak age of a first psychotic episode, and only 2 of 32 participants scoring above the screening threshold met formal criteria for CHR. Despite a few studies reporting on elevated levels of CHR symptoms in ASD and vice versa (Eussen et al. 2015; Solomon et al. 2011; Sprong et al. 2008), study samples are typically not screened concurrently for both conditions, and therefore very little is known about the predictive validity of prodromal symptoms in ASD. One 6-year follow-up study (de Wit et al. 2014) showed that out of 17 adolescents with ASD diagnosis and clinical high-risk, one individual (6%) had become psychotic, 5 (29%) were still considered at-risk, and 11 (65%) had remitted. Clearly additional follow-up studies in larger ASD/high-risk cohorts are required to address whether FTD constitutes a true risk factor for psychotic disorders in ASD.

Despite a global increase in overall FTD in our study, specific K-FTDS subtypes such as ‘Loose Associations’ and ‘Illogical Thinking’ did not differentiate between individuals with ASD and controls, which is inconsistent with the large effect sizes reported for these measures in previous studies (Solomon et al. 2008; van der Gaag et al. 2005). The only subtype that differed between groups was ‘Incoherence’, which denotes utterances that are difficult to understand due to insufficient organization. However, all effect sizes were small. A closer look at K-FTDS ratings across studies suggests that the average ratings in our study were quite low compared to the study by van der Gaag et al., possibly due to inclusion of a more mildly affected, slightly older ASD group and a lower utterance count. In terms of age, our study sample was similar to the Solomon et al. sample and FTD ratings for ASD were also highly comparable. However, FTD ratings for the control group in the Solomon study were virtually absent, whereas our control group did show some variation across FTD subtypes, and may therefore have been representative of a broader population. In addition, our sample sizes for both groups were roughly two-to-three times larger than in the two previously conducted studies, although the van der Gaag study also included an additional clinical comparison group diagnosed with MCDD. Interestingly, the MCDD group did not differ from the other ASD group on K-FTDS ratings.

To complement the objective measurement of FTDs, a subjective measure of FTD was also included in this study: the Odd Speech subscale of the Schizotypal Personality Questionnaire. For this measure we did find a significant group difference in the expected direction. Although the use of self-report questionnaires in individuals with ASD is sometimes scrutinized, the Odd Speech items are rather concrete and ask for both personal and observer qualifications of the subject’s speech difficulties. The fact that they are worded so that subjects also report on external corroboration of these symptoms probably indicates why the subscale can be measured quite reliably (Raine 1991), and why it has previously been used as a measure of FTD in patients with schizophrenia (Badcock et al. 2011). Training and rating procedures of K-FTDS are very time consuming and difficult to complete during clinical assessment (de Bruin et al. 2007), and therefore it is worth investigating alternative FTD measures that require less time and effort and may be more suitable for application in clinical or research settings where limited resources are available. Even though continuous measures for Odd Speech and K-FTDS were not significantly correlated, the binary variables (based on clinical cut-offs) were (Odd Speech − FTD Total: *ϕ* = 0.38, *p* ≡ .01). Although this suggests that both measures are only moderately tapping into the same construct, it is not uncommon for informant- and performance-based tasks measuring similar constructs to show limited correlations (Toplak et al. 2013). Clearly, different types of rater-bias and test impurity can dampen the strength of these monotrait-heteromethod correlations, possibly because one or several mediating variables are unaccounted for. However, both methods may also provide complementary information on the same underlying latent construct. Therefore, our first recommendation is to further investigate the reliability and validity of the Odd Speech subscale as a proxy for FTD in a multitrait-multimethod matrix and second, to establish whether it can explain additional variance in prediction models of psychosis, for example in CHR samples.

Despite robust findings of executive dysfunction in ASD, the literature on this topic is vast and riddled with conflicting findings. For example, it has been claimed that response inhibition is the only core executive function not impaired in ASD (Russo et al. 2007). However, a recent meta-analysis on prepotent response inhibition and interference control in ASD concluded that the evidence suggests otherwise (Geurts et al. 2014). Substantial inconsistencies have also been highlighted for working memory and cognitive flexibility performance in ASD (de Vries and Geurts 2014; de Vries et al. 2015), although rigorous meta-analyses for these constructs are currently lacking in the literature. Additionally, executive dysfunctions tend to have limited discriminative value across psychiatric classifications. Enriching between-group analyses with within-group associations may therefore be better suited to help identify specific associations between cognitive markers and heterogeneous behavioral phenotypes.

By applying this strategy, we were able to detect an association between executive functioning and FTD within the ASD group. Lower verbal working memory performance significantly predicted higher levels of FTD in both linear and logistic models and for both subjective and objective FTD in the latter. This suggests that individuals with ASD who experience verbal working memory difficulties may be particularly vulnerable for developing subsequent FTD symptoms. The task used (CELF-NR) is a digit span task, which (along with other distractibility tasks) has previously been associated with FTD in children with ADHD, but not in children with schizophrenia (Caplan et al. 2001). Given the high comorbidity of ADHD and ASD symptoms in general, it is possible that the association was mostly reflecting increased distractibility in some of our individuals with ASD. This would subsequently limit the capacity to encode and retrieve verbal information in/from working memory and thereby reduce reproduction of the presented stories in the story game, for example. However, the absence of similar associations for visuospatial working memory, as well as for the other non-verbal executive functioning parameters, and the relative preserved verbal skills which were not correlated to FTD in our ASD sample, all strengthen the notion that executive control over verbal processing is key to understanding FTD in ASD. Furthermore, Docherty (Docherty 2012) also found that verbal working memory (digit span task) predicted FTD in adults with schizophrenia. Although this does not directly implicate an increased risk for schizophrenia in ASD individuals with FTD, it could entail that the combination of clinical levels of FTD and verbal working memory problems poses a potential risk factor for psychotic episodes in ASD.

Two known studies have also directly addressed relations between executive functioning and FTD in ASD. Both indicated that prepotent response inhibition in ASD was significantly associated with FTD, respectively for K-FTDS—Illogical thinking (Solomon et al. 2008) and SPQ – Disorganization (= Odd Speech + Eccentric Behavior) (Barneveld et al. 2013). However, these studies consisted of smaller samples and correlational analyses were conducted with similar but slightly different parameters for only one executive functioning measure. Given the hypothesized dependent nature of FTD, regression analyses can provide better clues as to the relative impact of executive functions on FTD.

Two main limitations of our study need to be highlighted. Although we were able to increase sample size and include additional cognitive parameters and analyses compared to previous FTD studies in ASD samples, our study was still too limited to include additional predictors from other relevant neurocognitive domains, such as attention and language, which we recommend for future studies. An additional limitation is the use of cross-sectional data, which prohibits any inferences about the causal nature of any associations under investigation.

To conclude, the current study aimed to investigate relations between executive functions and FTD in children and adolescents with ASD and matched typically developing controls. In sum, we found evidence for an increased prevalence of FTD and a significant negative association between verbal working memory skills and FTD, which may overrule the potential impact of response inhibition or cognitive flexibility on disorganized speech. We therefore suggest that poor verbal working memory skills may predispose some children and adolescents with autism to develop thought disorder and advise researchers in the field to shift their focus from solely on executive control to include the role of executive verbal processing skills in relation to FTD in this target population.

## Electronic supplementary material

Below is the link to the electronic supplementary material.

Supplementary material 1 (DOCX 123 KB)
