# Supplementary material for: Formal Thought Disorder and Executive Functioning in Children and Adolescents with Autism Spectrum Disorder: Old Leads and New Avenues
Source: J Autism Dev Disord. 2017 Mar 24;47(6):1756–68. doi: 10.1007/s10803-017-3104-6 (PMC5432602; doi:10.1007/s10803-017-3104-6)
Supplement: Supplementary file 1 — Supplementary material 1 (DOCX 123 KB) [file 10803_2017_3104_MOESM1_ESM.docx]

**Supplemental Table.** Pairwise Spearman correlations of executive functions with global group characteristics and FTD

|  | 1 | 2 | 3 | 4 | 5 | 6 |  | 7 | 8 | 8a | 8b | 9 |  | 10 | 11 | 12 | 13 |
| --- | --- | --- | --- | --- | --- | --- | --- | --- | --- | --- | --- | --- | --- | --- | --- | --- | --- |
| **Executive functioning** |  |  |  |  |  |  |  |  |  |  |  |  |  |  |  |  |  |
| 1. verbal working memory |  | **.338^*^** | **-.212** | .157 | -.025 | -.101 |  | **.305^*^** | .167 | .083 | **.220** | -128 |  | -.123 | -.066 | **-.217** | -.158 |
| 2. visuo-spatial working memory | **.360** |  | **-.417^*^** | **.408^*^** | **-.365^*^** | **-.391^*^** |  | **.575^*^** | **.289^*^** | .139 | **.339^*^** | **-.204** |  | -.134 | -.084 | -.075 | -.183 |
| 3. response inhibition (speed) | -.212 | **-.375** | **^*^** | -.183 | **.331^*^** | .188 |  | **-.464^*^** | -.009 | .033 | -.031 | .142 |  | .125 | .090. | .162 | .152 |
| 4. response inhibition (accuracy) | .236 | **.426^*^** | **-.347** |  | -.176 | **-.222** |  | **.381^*^** | **.262^*^** | -.107 | **.295^*^** | .087 |  | -.168 | -.461 | -.102 | **-.240** |
| 5. cognitive flexibility (speed) | .085 | -.237 | .192 | -.179 |  | .135 |  | **-.413^*^** | .014 | .000 | -.010 | -.028 |  | -.010 | -.009 | -.034 | .070 |
| 6. cognitive flexibility (accuracy) | -.036 | **-.305** | .207 | -.152 | .228 |  |  | **-.342^*^** | **-.247** | -.018 | **-.220** | .032 |  | -.100 | -.128 | .001 | .136 |
| **Group Characteristics** |  |  |  |  |  |  |  |  |  |  |  |  |  |  |  |  |  |
| 7. age | .118 | **.430^*^** | **-.369^*^** | **.321** | -.250 | **-.435^*^** |  |  | -.026 | -.087 | .054 | -.015 |  | -.133 | -.130 | .006 | -.146 |
| 8. FSIQ | **.305** | **.490^*^** | -.208 | -.254 | -.161 | **-.306** |  | .036 |  | **.776^*^** | **.796^*^** | .079 |  | -.063 | -.060 | -.017 | -.100 |
| 8a. Verbal | .199 | .199 | -.088 | .056 | -.134 | -181 |  | -.012 | **.825^*^** |  | **.268^*^** | .197 |  | -.089 | -.091 | -.021 | -.073 |
| 8b. Performal | **.332** | **.617^*^** | -.268 | **.354** | -.157 | **-.333** |  | .097 | **.829^*^** | **.381^*^** |  | -.070 |  | -.004 | .010 | -.040 | -.074 |
| 9. AQ - Total | -.247 | -.166 | -.043 | -.020 | -.252 | -.221 |  | -.001 | -.047 | .007 | -.073 |  |  | .191 | .124 | .176 | **.304^*^** |
| **Formal thought disorder** ^1^ |  |  |  |  |  |  |  |  |  |  |  |  |  |  |  |  |  |
| 10. K-FTDS - Total FTD | -.242 | -.175 | .205 | -.140 | -.012 | -.123 |  | -.181 | -.165 | -.136 | -.155 | .103 |  |  | **.943^*^** | **.361^*^** | .167 |
| 11. K-FTDS - Illogical thinking | -.164 | -.102 | .106 | -.054 | -.047 | -.162 |  | -.168 | -.157 | -.147 | -.119 | -.032 |  | **.904^*^** |  | .118 | .132 |
| 12. K-FTDS - Incoherence | -.238 | -.070 | .265 | -.214 | .004 | -.049 |  | -.009 | -.122 | -.091 | -.162 | .257 |  | **.448^*^** | .167 |  | **.217** |
| 13. SPQ - Odd Speech | **-.400^*^** | -.183 | .135 | -.080 | .134 | .031 |  | -.231 | -.133 | -.089 | -.166 | .029 |  | .226 | .268 | .180 |  |

**bold** = p<.05; ^*^ = p<.01; coefficients for total group (*N*=106) are displayed above the diagonal, below the diagonal shows coefficients for the ASD group only (*N*=50)

^1^K-FTDS ratings represent errors divided by utterances per minute; subscales for loose associations and poverty of content were not included due to low frequencies.
